# Electrophysiological and morphological changes in colonic myenteric neurons from chemotherapy‐treated patients: a pilot study

**DOI:** 10.1111/nmo.12795

**Published:** 2016-02-22

**Authors:** S. E. Carbone, V. Jovanovska, S. J. H. Brookes, K. Nurgali

**Affiliations:** ^1^ Centre for Chronic DiseaseCollege of Health and BiomedicineVictoria UniversityMelbourneVICAustralia; ^2^ Discipline of Human Physiology and Centre for NeuroscienceFlinders UniversityAdelaideSAAustralia

**Keywords:** chemotherapy, colorectal cancer, electrophysiology, enteric neurons, human

## Abstract

**Background:**

Patients receiving anticancer chemotherapy experience a multitude of gastrointestinal side‐effects. However, the causes of these symptoms are uncertain and whether these therapeutics directly affect the enteric nervous system is unknown. Our aim was to determine whether the function and morphology of myenteric neurons are altered in specimens of the colon from chemotherapy‐treated patients.

**Methods:**

Colon specimens were compared from chemotherapy‐treated and non‐treated patients following colorectal resections for removal of carcinoma. Intracellular electrophysiological recordings from myenteric neurons and immunohistochemistry were performed in whole mount preparations.

**Key Results:**

Myenteric *S* neurons from chemotherapy‐treated patients were hyperexcitable; more action potentials (11.4 ± 9.4, *p* < 0.05) were fired in response to depolarising current pulses than in non‐treated patients (1.4 ± 0.5). The rheobase and the threshold to evoke action potentials were significantly lower for neurons from chemotherapy‐treated patients compared to neurons from non‐treated patients (*p* < 0.01). Fast excitatory postsynaptic potential reversal potential was more positive in neurons from chemotherapy‐treated patients (*p* < 0.05). An increase in the number of neurons with translocation of Hu protein from the cytoplasm to the nucleus was observed in specimens from chemotherapy‐treated patients (103 ± 25 neurons/mm^2^, 37.2 ± 7.0%, *n* = 8) compared to non‐treated (26 ± 5 neurons/mm^2^, 11.9 ± 2.7%, *n* = 12, *p* < 0.01). An increase in the soma size of neuronal nitric oxide synthase‐immunoreactive neurons was also observed in these specimens.

**Conclusions & Inferences:**

This is the first study suggesting functional and structural changes in human myenteric neurons in specimens of colon from patients receiving anticancer chemotherapy. These changes may contribute to the causation of gastrointestinal symptoms experienced by chemotherapy‐treated patients.

**Key Points**

- This is the first electrophysiological study of human enteric neurons in a pathological condition.
- This study aimed to investigate the effects of anticancer chemotherapy on functional and morphological properties of human myenteric neurons.
- Intracellular electrophysiology combined with morphological identification of recorded neurons and immunohistochemistry were used to characterize myenteric neurons in fresh colon specimens from colorectal cancer patients treated and untreated with chemotherapeutic agents.
- The results of this study demonstrated hyperexcitability of myenteric *S* neurons, increase in the number of neurons with translocation of Hu protein from the cytoplasm to the nucleus, and increase in the soma size of neuronal nitric oxide synthase‐immunoreactive neurons from chemotherapy‐treated patients.

## Introduction

Colorectal cancer (CRC) is the second most commonly diagnosed cancer and is a major cause of cancer‐related deaths worldwide. Chemotherapy alone, or in combination with radiotherapy, is given before or after surgery to most patients. Currently, the standard first‐line chemotherapy of metastatic CRC is 5‐fluorouracil (5‐FU) combined with folinic acid plus either oxaliplatin (FOLFOX) or irinotecan (FOLFIRI).^1^ Although these drugs increase survival rate and reduce the risk of disease progression, both have acute and long‐term toxicities leading to a wide spectrum of side‐effects. Symptoms such as pain, paresthesia (tingling or numbness), cold‐induced dysesthesia (burning sensations), and a general loss of sensation^2^, ^3^, ^4^, ^5^, ^6^ have been attributed to peripheral neuropathies resulting from the neurotoxic effects of chemotherapeutic drugs. Severe gastrointestinal side‐effects include nausea, vomiting, constipation, and diarrhoea.^7^, ^8^, ^9^ Gastrointestinal toxicity is one of the main reasons for dose limitation of chemotherapy, often reducing the efficacy of anticancer treatment. Chronic gastrointestinal side‐effects can persist for more than 10 years post treatment, greatly affecting patients' quality of life.^10^ The traditional view is that gastrointestinal side‐effects of ant‐cancer drugs are due to mucosal damage.^11^ Although mucosal damage undoubtedly plays a significant role in the acute symptoms associated with chemotherapeutic treatment, the persistence of gastrointestinal symptoms suggests that chemotherapy may damage the gastrointestinal innervation. The enteric nervous system controls many major functions of the gut, including motility, blood flow, secretion and absorption of nutrients, electrolytes, and water.^12^ The morphology and functions of enteric neurons are compromised in various gastrointestinal pathologies.^13^ Because anticancer chemotherapeutics cause widespread peripheral neuropathy, we hypothesized that gastrointestinal side‐effects associated with chemotherapy may result from damage to the enteric nervous system.

Few studies have examined the effects of anticancer chemotherapies on the enteric nervous system. We have previously shown changes in colonic motility and reduction in the total number of neurons within the myenteric plexus in mice treated *in vivo* with the anticancer chemotherapeutic oxaliplatin.^14^ Similar results were found in the colon of rats treated with another platinum‐based chemotherapeutic agent, cisplatin.^15^ In addition, cisplatin‐treated rats had reduced gastric motility.^15^, ^16^, ^17^

Immunohistochemical methods have been extensively used to document changes in the neurochemical coding and morphology of human enteric neurons under a variety of conditions.^18^, ^19^, ^20^, ^21^ Electrophysiological recordings of human enteric neurons in freshly dissected preparations reveal functional properties, but have only been reported in two published studies to date, both from specimens of the colon.^22^, ^23^ Studies based on these techniques can demonstrate changes in certain conditions, such as in inflammation, where enteric neurons become hyperexcitable.^24^, ^25^

This study aimed to investigate the effects of anticancer chemotherapeutics on human myenteric neurons. Using electrophysiology combined with immunohistochemistry, the functional properties of morphologically identified myenteric neurons were compared in colon specimens from chemotherapy‐treated versus non‐treated patients for the first time.

## Materials and Methods

Specimens of the human colon were provided by the Victorian Cancer Biobank (number of individual patients *N* = 13) and Flinders Medical Centre (*N* = 8). All studies were approved by the Victoria University Human Research Ethics and Southern Adelaide Clinical Research Ethics Committees and have been performed in accordance with the ethical standards laid down in the 1964 Helsinki Declaration and its later amendments. Prior to surgical removal of non‐obstructive carcinoma, written informed consent was obtained from all patients. Fresh specimens were delivered after the surgery in Roswell Park Memorial Institute (RPMI) culture medium or Krebs solution at 4 °C. Of all 21 specimens, nine were from patients who had received chemotherapeutic treatments. Due to the limited number of samples available, all specimens from chemotherapy‐treated patients were combined in a single chemotherapy‐treated group. Patients received 5‐Fluorouracil alone (5‐FU, *N* = 1), combined FOLFOX regimen (Folinic acid, 5‐FU, and oxaliplatin, *N* = 4) for 6 cycles and neoadjuvant 5‐FU in combination with radiotherapy treatment (*N* = 4). Control specimens were obtained from patients who had not received chemotherapy or radiotherapy prior to surgery (*N* = 12, termed *non‐treated patients*). The age of patients at the time of surgery ranged between 39 and 89 years. Non‐treated patients averaged 72.9 ± 3.0 years (10 male, 2 female) and chemotherapy‐treated patients averaged 57.4 ± 4.7 years (7 male, 2 female). Specimens from non‐treated patients (distal colon: 7, proximal colon: 5) as well as specimens from chemotherapy‐treated patients (distal colon: 8, proximal colon: 1) were predominantly from the distal colon. Aside from two specimens (both from non‐treated patients), all segments of the colon were from regions distal to the tumor, but the distances from the tumor were not made available.

The methods used in this study have been described previously.^22^ Briefly, full thickness specimens were placed in Krebs solution at room temperature (mM: NaCl 118, KCl 4.6, CaCl_2_ 3.5, MgSO_4_ 1.2, NaH_2_PO_4_ 1, NaHCO_3_ 25, D‐Glucose 11, bubbled with 95% O_2_ and 5% CO_2_) and pinned in a Sylgard‐lined Petri dish (Dow Corning, Midland, MI, USA) to make a flat sheet preparation for dissection. The serosa, fat, mesentery, mucosa, and submucosa were removed using sharp dissection techniques and the myenteric plexus was exposed by peeling away the circular muscle layer with forceps. The Krebs solution was changed at least every 15 min.

### Intracellular recording

Nine specimens were used for intracellular electrophysiology analysis (non‐treated: five specimens, chemotherapy‐treated: four specimens). Specimens were repinned in a recording chamber with 50 *μ*m gold plated, tungsten pins. Myenteric ganglia were identified by creamy‐white pigmentation. Extra pins (20 *μ*m diameter wire) were added nearby to stabilize ganglia for recording. The chamber was mounted on a Zeiss Axiovert‐200 inverted microscope (Zeiss, Oberkochen, Germany). To recover from dissection, the preparation was superfused with Krebs solution containing 1 *μ*M atropine and 1 *μ*M nicardipine (35 °C) for 1 h.^26^ Conventional glass micropipettes were used to impale neurons. They contained 5% 5,6‐carboxyfluorescein in 20 mM Tris buffer in 1 M KCl (pH 7.0)^26^ and had resistances of 100–150 MΩ. An Axoclamp 2B amplifier (Axon Instruments, Foster City, CA, USA) was used for recordings; signals were digitized at 1–10 kHz by a Digidata 1440A interface (Molecular Devices, Sunnyvale, CA USA) and stored on a computer using PClamp 10.0 (Molecular Devices). To identify the morphology of the impaled cell, carboxyfluorescein was injected by iontophoresis using hyperpolarizing current pulses (0.5 nA, 0.2 s duration at 2.5 Hz) for 2 min. Cells labeled in this way could be visualized *in situ* using fluorescence.^22^, ^26^ Only neurons that were adequately filled with carboxyfluorescein and had resting membrane potentials (RMPs) more negative than −40 mV were analyzed. Input resistance (R_in_) was calculated from intracellular hyperpolarizing current pulses (500 ms, 100–500 pA). A tungsten stimulating electrode (10–50 *μ*m tip diameter), connected to an ISO‐Flex stimulator (AMPI, Jerusalem, Israel), was either positioned between identified ganglia and 1 mm circumferential to the impaled cell. Axon tracts were stimulated by a single‐shot stimulus (0.4 ms; 10–60 V), so that fast excitatory postsynaptic potentials (fEPSPs) could be recorded in the impaled cell. Slow excitatory postsynaptic potentials were rarely evoked even by trains of stimuli and were therefore not studied. Axograph X software was used to analyze data. Following recording, preparations were fixed in Zamboni's fixative (2% formaldehyde and 0.2% picric acid) overnight at 4 °C and processed for immunohistochemistry.

### Immunohistochemistry

Preparations were similarly dissected for immunohistochemistry. They were stretched to a maximum length (nicardipine [3 *μ*M] was added to the Krebs solution to facilitate maximal stretching), then fixed in Zamboni's fixative (4 °C for 48 h), cleared using dimethylsulfoxide (3 times for 10 min), then washed with phosphate buffered saline (PBS, 3 times for 10 min). Specimens were incubated in primary antibodies: goat antineuronal nitric oxide synthase (nNOS, 1 : 500, NB100‐858; Novus Biologicals, Littleton, CO, USA), mouse anti‐Hu (1 : 500, A21271; Molecular Probes, Eugene, OR, USA) for 48 h, rinsed in PBS and incubated in secondary antibodies: DyLight 405 donkey antigoat and antimouse Alexa Fluor 594 (1 : 200; Jackson Immunoresearch Laboratories, West Grove, PA, USA) for 4 h. Tissues were viewed on an IX71 Olympus microscope (Olympus, Tokyo, Japan) or an Eclipse Ti confocal microscope (Nikon, Tokyo, Japan).

Images of four randomly selected ganglia, showing myenteric neurons immunoreactive (IR) for Hu and nNOS, were captured by confocal microscopy at 20× magnification. The number of neurons within each image was counted to calculate the total number of neurons/mm^2^. The soma‐dendritic area (*μ*m^2^) of nNOS‐IR neurons was measured by tracing neuronal profiles, using Image J software (NIH, Bethesda, MD, USA). The average size of neurons was calculated from 10 cells per image.

### Drugs

All drugs used in this study and 5,6‐carboxyfluorescein were purchased from Sigma‐Aldrich (Castle Hill, NSW, Australia). Both nicardipine and atropine were dissolved in sterile water and stored at 10^−2^ M.

### Statistics

For electrophysiology results, where the properties of neurons were compared, *n* is the number of cells and *N* is the number of tissue samples, which was equivalent to the number of patients. In some instances, more than one neuron was recorded from a single patient; values are presented as means for all cells ± SD. For immunohistochemistry results, *n* and *N* are the same; values are represented as means ± SEM to demonstrate how the sample mean represents the population mean. Results were compared using an unpaired *t*‐test without variance correction. Differences were considered statistically significant at *p* < 0.05. All authors had access to the study data and had reviewed and approved the final manuscript.

## Results

### The effects of chemotherapeutic treatment on the electrophysiological properties of colonic myenteric neurons

Enteric neurons can be classified as *S* or *AH* cells based on their electrophysiological properties.^12^ *S* cells had no visible inflection on the falling phase of their action potentials, lacked a long after‐hyperpolarization following a single action potential and showed fast EPSPs in response to focal electrical stimulation of nearby nerve tracts. A long after‐hyperpolarization was recorded in only one neuron; this neuron also had an inflection on the falling phase of its action potential; features identifying it as an *AH* type II enteric neurons. Three additional neurons had inflections on the falling phase of their action potentials but lacked a long after‐hyperpolarization. These neurons were multiaxonal dendritic Dogiel type II neurons. Due to the scarcity of *AH* neurons, only *S* neurons confirmed by the presence of a fast EPSP and uniaxonal morphology were analyzed in this study.

**Figure 1:**
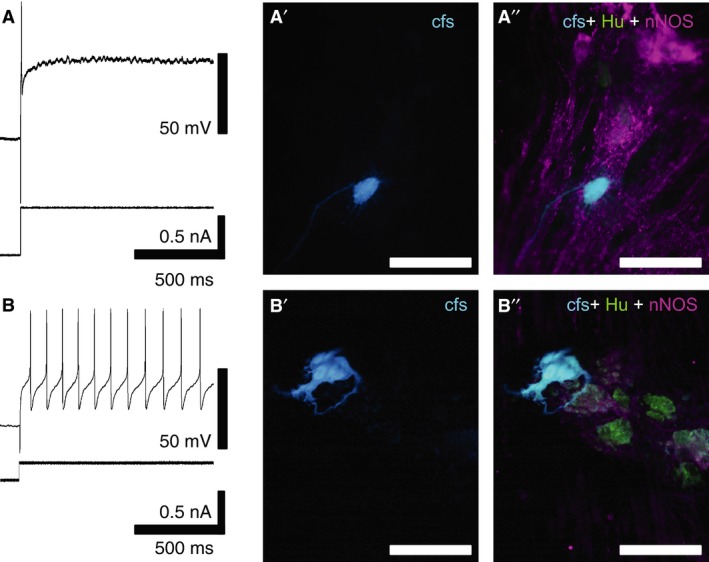
Electrophysiological properties of colonic myenteric neurons from non‐treated and chemotherapy‐treated patients. Action potentials were evoked with depolarizing current pulses. (A) A neuron from a colonic specimen of a non‐treated patient fired a single action potential in response to a depolarizing current. (A') Intracellular injection of carboxyfluorescein during recording confirmed that this cell had Dogiel type I, uniaxonal morphology. (A”) Immunohistochemistry demonstrated that this neuron was Hu‐immunoreactive but did not contain nNOS immunoreactivity. (B) A neuron from a chemotherapy‐treated patient fired multiple action potentials in response to a smaller depolarising current pulse. (B') Intracellular injection of carboxyfluorescein confirmed the neuronal morphology of the cell. (B”) This neuron was Hu‐immunoreactive, but not nNOS‐immunoreactive, scale bar = 100 *μ*m.

The RMP and R_in_ of *S* type I myenteric neurons in colonic specimens from non‐treated (RMP: −59.7 ± 11.5 mV, R_in_: 100.5 ± 78.2 MΩ, *n* = 5, *N* = 5) or chemotherapy‐treated (RMP: −57.3 ± 1.6 mV; R_in_: 132.7 ± 125.6 MΩ, *n* = 5, *N* = 4) patients were not significantly different. Myenteric *S* neurons fired action potentials when stimulated with depolarizing current pulses (Fig 1A and B). Injection of depolarizing currents (500–800 pA, 1.0 s) into neurons from non‐treated patients evoked one to two action potentials. The number of action potentials averaged over the first 1 s period of depolarization was 1.4 ± 0.5 (*n* = 5, *N* = 5, example Fig 1A). *S* neurons in colonic specimens from chemotherapy‐treated patients fired continuous bursts of action potentials in response to depolarizing current pulses (20–500 pA, 1–5 s). The number of action potentials in 1 s averaged 11.4 ± 9.4 (*n* = 5, *N* = 4). Thus, S cells from chemotherapy‐treated patients were significantly more excitable than those from non‐treated patients (*p* < 0.05). The patients' ages used for the analysis of neuronal excitability were: 86, 62, 79, 74, 62 in the non‐treated group and 79, 78, 60, 47 in chemotherapy‐treated group. Preparations were from the distal (*N* = 3) and ascending (*N* = 2) colons in the non‐treated group and all (*N* = 5) from the distal colon in the chemotherapy‐treated group; all samples were distal to non‐obstructive carcinoma. Thus, neuronal excitability did not appear to depend on the age of patients or the location of the cells, although given the low numbers of patients studied it is not possible to reach a firm conclusion. Carboxyfluorescein filling identified that all of the neurons included in this analysis were uniaxonal Dogiel type I neurons (Fig 1A'–B”).

**Figure 2:**
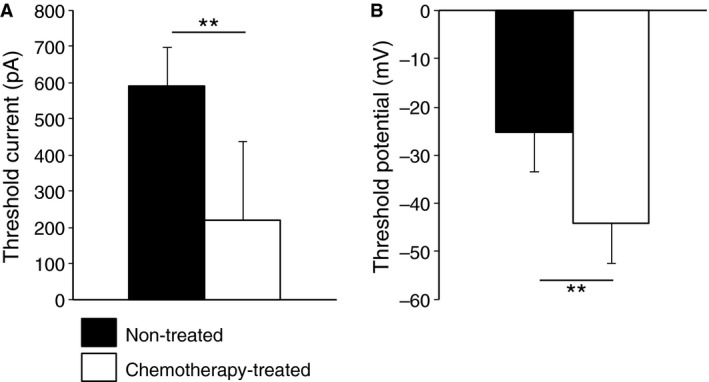
Myenteric *S* neurons from colonic specimens of chemotherapy‐treated patients are hyperexcitable compared to neurons from non‐treated patients. (A) Neurons from non‐treated patients required a larger depolarizing current to evoke action potentials (rheobase) compared to neurons from chemotherapy‐treated patients. (B) The action potential threshold was less negative in neurons from non‐treated patients (*n* = 5, *N* = 5) compared to neurons from chemotherapy‐treated patients (*n* = 5, *N* = 4, **p < 0.01, data expressed as mean ± SD).

The threshold currents required to evoke an action potential (rheobase) were calculated when the cell was manually clamped at −70 mV. The rheobase of neurons from chemotherapy‐treated patients (221 ± 215 pA, *n* = 5, *N* = 4) was significantly lower than non‐treated patients (591 ± 106 pA, *n* = 5, *N* = 5, *p* < 0.01, Fig. 2A). Similarly, the threshold potentials at which action potentials were evoked were more negative in neurons from chemotherapy‐treated patients (−44.2 ± 8.2 mV *n* = 5, *N* = 4) compared to neurons from non‐treated patients (−25.3 ± 8.2, *n* = 5, *N* = 5, *p* < 0.01, Fig. 2B). Anodal‐break action potentials did not occur in neurons from either group.

**Figure 3:**
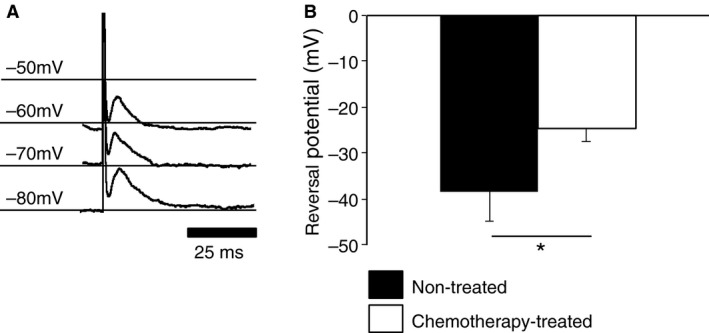
The reversal potentials of fast excitatory postsynaptic potentials (fEPSPs) were less negative in neurons from chemotherapy‐treated patients. (A) The holding potential of the cell was set by manual current clamp; fast EPSP amplitude increased at hyperpolarized potentials. (B) The extrapolated reversal potential of fEPSP in neurons from non‐treated patients (*n* = 4, *N* = 4) was more negative than in cells from chemotherapy‐treated (*n* = 3, *N* = 3) patients (*p < 0.05).

Fast excitatory postsynaptic potentials, were present, either evoked or spontaneous, in all *S* neurons. Their amplitudes were compared with the cell manually clamped at −70 mV. Differences were not significant between the amplitudes of spontaneous fEPSPs recorded at −70 mV in neurons from chemotherapy‐treated (average = 6.8 ± 2.3 mV [6.4 mV, 4.7 mV, 9.2 mV] *n* = 3, *N* = 3) and non‐treated patients (average = 3.9 ± 1.5 mV [4.3 mV, 5.9 mV, 2.6 mV, 3.0 mV] *n* = 4, *N* = 4, *p* = 0.1). Average intervals between spontaneously occurring fEPSPs in neurons from chemotherapy‐treated (average = 0.7 ± 0.1 s [0.7 s, 0.6 s, 0.8 s] *n* = 3, *N* = 3) and non‐treated (average = 2.7 ± 2.4 s [0.5 s, 4.9 s, 4.6 s, 0.9 s] *n* = 4, *N* = 4) patients were also not significantly different (*p* = 0.2). The reversal potential for fEPSPs was calculated by plotting evoked fast EPSP amplitude at a range of holding potentials (Fig. 3A). Fast EPSP reversal potential was more positive for neurons from chemotherapy‐treated patients (average = −24.5 ± 2.9 mV [−26.3 mV, −26.1 mV, −21.2 mV], *n* = 3, *N* = 3) than in myenteric neurons from non‐treated patients (average = −38.4 ± 6.4 mV [−32.1 mV, −46.6 mV, −40.1 mV, −34.7 mV], *n* = 4, *N* = 4, *p* < 0.05, Fig. 3B). The mean amplitude of evoked fEPSPs, measured at a holding potential of −70 mV, tended to be smaller in neurons from non‐treated patients (average = 7.2 ± 3.2 mV [4.9 mV, 11.9 mV, 5.9 mV, 6.0 mV] *n* = 4, *N* = 4) compared to neurons from chemotherapy‐treated patients (average = 14.5 ± 5.1 mV [20.4 mV, 12.0 mV, 11.1 mV] *n* = 3, *N* = 3), but this did not reach significance with the available sample sizes (*p* = 0.06).

### Morphological changes in myenteric neurons from chemotherapy‐treated patients

**Figure 4:**
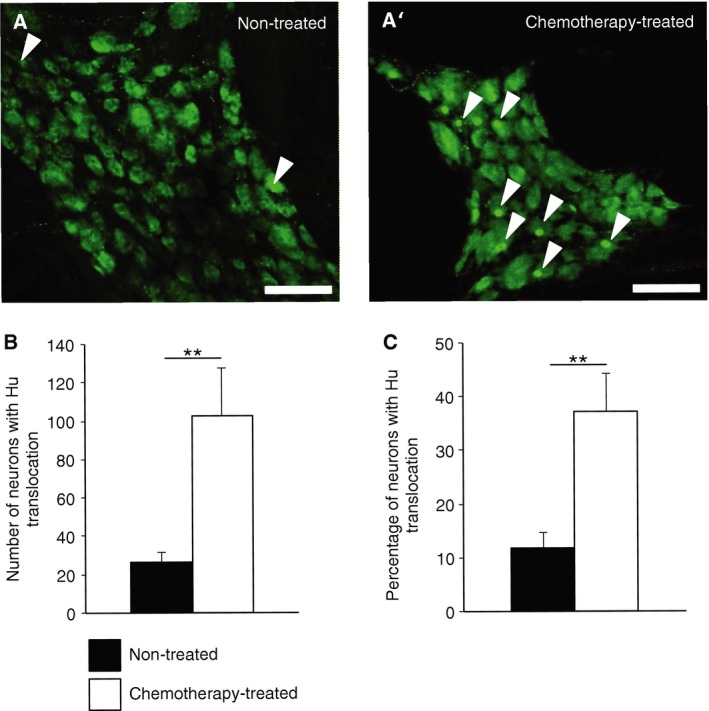
Whole mount preparations of myenteric neurons from non‐treated and chemotherapy‐treated patients labeled with the pan‐neuronal marker Hu. (A and A') Translocation of Hu protein to the cell nuclei was noted in some cells (arrowheads) (scale bar = 100 *μ*m). The total number of neurons counted per field of view with Hu translocation (B) and the overall percentage of neurons with Hu translocation (C) were significantly greater in the myenteric plexus from colonic specimens of chemotherapy‐treated patients (*n* = 8, *N* = 8) compared to non‐treated patients (*n* = 9, *N* = 9)(**p < 0.01).

**Figure 5:**
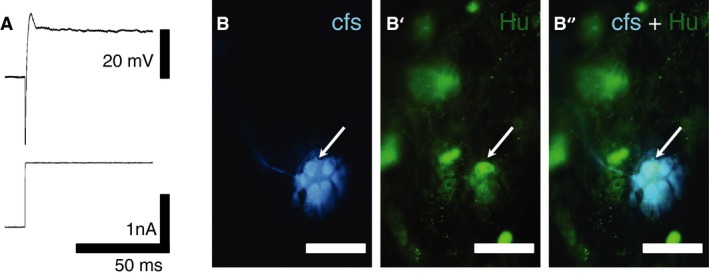
Intracellular recording from a myenteric neuron with Hu‐translocation. (A) Depolarization with a large current pulse failed to evoke an action potential in this neuron. (B) Intracellular injection of carboxyfluorescein confirmed the neuronal morphology. The arrow indicates the nucleus. (B' and B”) Immunohistochemical labeling revealed translocation of Hu protein to the nucleus (arrows) (scale bar = 50 *μ*m). Images were viewed on a Nikon confocal microscope (Eclipse Ti) with 20 × objective.

Preparations were labeled as whole mounts with a pan‐neuronal marker, Hu antibody (Fig. 4A and A'). The total number of Hu‐IR neurons/mm^2^ was similar in chemotherapy‐treated patients (263.6 ± 17.8 neurons/mm^2^, *n* = 8) and non‐treated patients (216.1 ± 26.2 neurons/mm^2^, *n* = 12, *p* = 0.1). In most cells, the Hu antibody evenly labeled the cytoplasm and nucleus of the cell but in some instances more intense staining of the nucleus was noted, suggesting translocation of Hu protein to the nucleus (Fig. 4A and A', arrowheads). The total number of neurons with Hu translocation (Fig. 4B) and the percentage of neurons with Hu translocation (Fig. 4C) were significantly greater in preparations from chemotherapy‐treated patients compared to non‐treated patients (chemotherapy‐treated: 103 ± 25 neurons/mm^2^, 37.2 ± 7.0%, *n* = 8; non‐treated: 26 ± 5 neurons/mm^2^, 11.9 ± 2.7%, *n* = 12, *p* < 0.01). The occurrence of Hu translocation appeared independent of age (linear regression analysis, data not shown). None of the neurons recorded electrophysiologically had detectable Hu translocation. However, two neurons (that were not included in the analysis) failed to generate action potentials in response to large depolarizing current pulses (>1 nA); both had intensely Hu‐labeled nuclei consistent with Hu translocation; these cells were both from non‐treated patients (Fig. 5).

**Figure 6:**
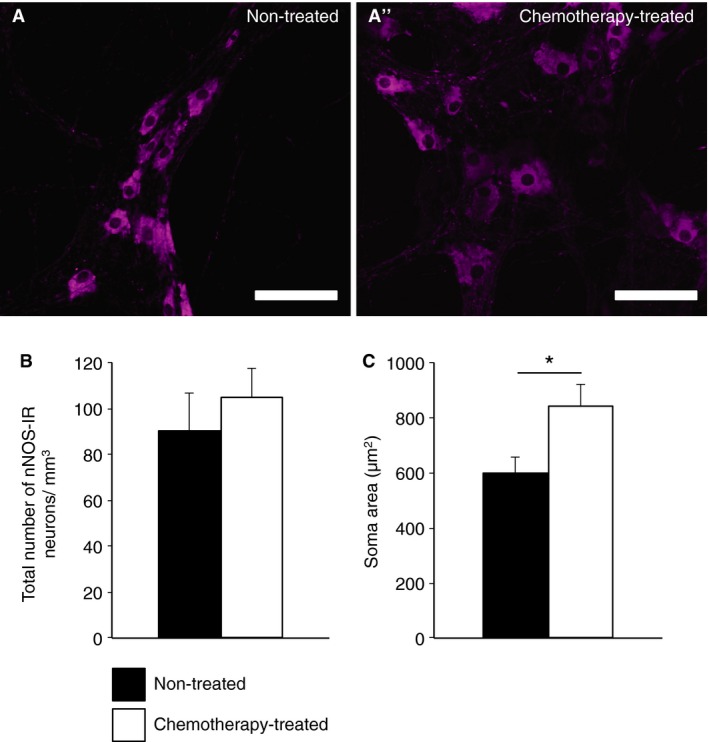
Nitrergic myenteric neurons in specimens of the colon from non‐treated and chemotherapy‐treated patients. (A and A') Neurons were labeled for with nNOS antibody in whole mount preparations (scale bar = 100 *μ*m). (B) The total number of nNOS‐immunoreactive neurons per field of view was not significantly different in the myenteric plexus of the colons from non‐treated (*n* = 9, *N* = 10) *vs* chemotherapy‐treated (*n* = 7, *N* = 10) patients. (C) The soma areas of nNOS‐immunoreactive neurons were significantly larger in colon specimens from chemotherapy‐treated patients (*n* = 4, *N* = 4) *vs* non‐treated patients (*n* = 8, *N* = 8) (*p < 0.05).

Preparations were also labeled with anti‐nNOS antibody, a marker for inhibitory motor neurons and some interneurons (Fig. 6A and A'). The number of myenteric neurons expressing nNOS, as well as their proportion in colon specimens from chemotherapy‐treated patients (104.6 ± 13.0 neurons/mm^2^, 41.5 ± 4.4%, *n* = 7), did not differ from specimens from non‐treated patients (90.4 ± 15.9 neurons/mm^2^_,_ 39.9 ± 4.6%, *n* = 9, *p* = 0.5, Fig. 6B). However, the soma‐dendritic area of nNOS‐IR neurons (measured from vertical projections) from chemotherapy‐treated patients (840.7 ± 79.8 *μ*m^2^, *n* = 4) was significantly greater than those from non‐treated patients (601.4 ± 53.2 *μ*m^2^, *n* = 8, *p* < 0.05, Fig. 6C). Hu translocation in nNOS‐IR neurons was not more frequent than in preparations from chemotherapy‐treated patients (20.8 ± 7.5, *n* = 5) compared to non‐treated patients (14.6 ± 2.5, *n* = 5, *p* = 0.5). Of the neurons in the electrophysiology analysis, 2/5 neurons from chemotherapy‐treated patients expressed detectable nNOS immunoreactivity; both neurons fired multiple action potentials in response to a depolarizing current pulse.

## Discussion

This study has provided evidence that the electrophysiological and morphological features of human myenteric neurons are altered in patients treated with chemotherapeutic agents. These changes include: (i) hyperexcitability of *S* neurons with uniaxonal Dogiel type I morphology, (ii) changes in the reversal potential of fast EPSPs, (iii) increased number and proportion of neurons with translocation of Hu protein from the cytoplasm into the nucleus, and (iv) increase in average soma size of nNOS‐IR neurons.

Our results demonstrated that the threshold for action potential firing was more negative in chemotherapy‐treated patients compared to non‐treated, suggesting greater excitability in the former group. In addition, the number of action potentials in response to a standardized current pulse was significantly greater in *S* neurons from chemotherapy‐treated patients compared to non‐treated patients. Furthermore, smaller depolarizing currents were needed to evoke an action potential when RMP was manually clamped at −70 mV. Thus, in combination, these data provide evidence of hyperexcitability of enteric neurons in chemotherapy‐treated patients. The excitability of *S* neurons must be determined by their complement of ion channels; these may be modulated by a variety of intracellular and extracellular factors. One possibility is that the increase in firing to depolarizing current pulses, lower threshold potential, and smaller rheobase were caused by a change in properties of voltage‐sensitive Na^+^ currents caused by chemotherapy. Previous studies reported chemotherapy‐induced hyperexcitability in dorsal root ganglion (DRG) sensory neurons.^27^ It has been proposed that metabolites of chemotherapeutic agents, such as oxalate from oxaliplatin, may modify the properties of voltage‐gated Na^+^ channels resulting in a prolonged open state,^28^ thus causing cellular hyperexcitability. Oxaliplatin modulates Na^+^ channel properties in nerve conduction studies especially in sensory nerve fibers in patients suffering from post‐chemotherapy neuropathy, although it tends to decrease excitability after long‐term exposure in contrast to this study where increased excitability of enteric neurons was evident.^29^, ^30^ In clinical studies, abnormalities in Na^+^ currents were detected in 78% of patients with chronic symptoms of chemotherapy‐induced peripheral neuropathy.^30^ While there is an evidence of hyperexcitability of sensory neurons and peripheral nerves after treatment with platinum‐based chemotherapy,^2^ peripheral sensory neuropathy associated with 5‐FU treatment is rare^31^ and has not been extensively studied. However, gastrointestinal dysmotility induced by 5‐FU treatment outlasts mucosal inflammation.^32^ Our study is the first demonstrating hyperexcitability of enteric neurons from patients treated with 5‐FU alone or in combination with oxaliplatin or radiotherapy. Enteric neuronal hyperexcitability, leading to long‐term intestinal dysfunction, has been demonstrated in animal models of intestinal inflammation.^24^, ^25^, ^33^ The hyperexcitability of enteric neurons observed in our study may contribute to the intestinal dysfunctions associated with chemotherapy. Approximately, 40% of patients receiving standard‐dose chemotherapy and 100% of patients receiving high‐dose chemotherapy for gastrointestinal cancers experience pain, bloating, diarrhea and/or constipation throughout the course of treatment.^34^ Gastrointestinal side‐effects are a predominant reason for dose limitation, presenting a constant challenge for efficient and tolerable treatment of CRC.^8^ Chronic gastrointestinal side‐effects can persist for more than 10 years after treatment is stopped, greatly affecting patients' quality of life.^10^

Our study also suggested that fast EPSPs in myenteric neurons from chemotherapy‐treated patients tend to be slightly larger in amplitude (although this was not significant with the small sample size). The reversal potential of electrically‐evoked fEPSPs was less negative in neurons from chemotherapy‐treated patients compared to controls. Both of these changes to cholinergic neurotransmission would be expected to increase excitability of enteric neural circuits. They are comparable with previously reported ectopic activity observed at motor and autonomic neuromuscular junctions after chemotherapy, which were attributed to the changes in Na^+^ channel properties.^28^ The mechanisms underlying changes in fast synaptic transmission in enteric neurons remain to be elucidated. They may result from direct effects of oxaliplatin or its metabolites on ion channels, or be indirectly mediated via other cells or mechanisms such as oxidative stress or inflammation. In the case of peripheral neuropathy, chemotherapeutics have multiple effects on neurons.^9^, ^35^

Hu immunolabeling has been used as a pan‐neuronal marker of the enteric nervous system in laboratory animals^36^, ^37^ and humans.^38^, ^39^ Typically, the cytoplasm and nucleus of neurons are uniformly labeled, with little or no staining of axons. In our study, Hu translocation from the cell body to the nucleus was observed as increased intensity of nuclear immunofluorescence. This phenomenon occurred in a higher proportion of neurons in the colon of chemotherapy‐treated patients than non‐treated patients. Hu proteins are a family of RNA‐binding proteins, expressed from the *Elavl (Elav‐like)* genes. *Elavl1*, which encodes HuR protein is expressed in a wide range of tissues,^40^ but *Elavl2*,* Elavl3*, and *Elavl4* (which encode HuB, HuC, and HuD proteins) are exclusively expressed in neurons.^41^ Hu proteins regulate post‐transcriptional gene expression through alternate splicing and RNA editing^42^, and stabilize mRNA by binding to uracil‐rich RNA sequences (AU and GU). Continuous shuttling of Hu proteins between the nucleus and cytoplasm has been implicated in their critical roles in control of nuclear export, stabilization, and cell survival.^43^ Their role in post‐transcriptional regulation adapts the proteome to changes in environmental conditions. Like other RNA‐binding proteins, Hu proteins change their expression level and subcellular location in response to stressful stimuli. Toxic neuropathy, associated with antiretroviral treatment, induced overexpression of HuD in DRG and spinal cord neurons.^44^ Altered intracellular localization of Hu proteins has been reported in response to DNA damaging agents such as ultraviolet light, heat shock, and actinomycin D.^45^, ^46^ Increased translocation of neuronal Hu proteins from the cytoplasm to the nucleus was observed in myenteric neurons after intestinal ischemia/reperfusion damage.^47^

Studies in the central nervous system demonstrated that Hu proteins promote the synthesis of the excitatory neurotransmitter glutamate^48^ and play important roles in neural plasticity, maintenance, and survival.^41^ In our study, myenteric neurons with Hu translocation to the nuclei failed to evoke action potentials, suggesting that intracellular localization of Hu proteins plays important role in cellular functioning.

In this study, nNOS‐IR cells in the colon samples from chemotherapy‐treated patients were larger than those in non‐treated patients. This is similar to our previous observations in chemotherapy‐treated mice.^14^ It was notable that Hu translocation, induced by chemotherapy, was less marked in nNOS‐IR enteric neurons than in cells that lacked nNOS. This suggests that nNOS‐expressing cells may be differentially affected by oxaliplatin treatment, with a tendency to increase in size rather than translocate Hu. Moreover, functional properties of these neurons were different. Unlike neurons with Hu translocation, nNOS‐IR neurons were hyperexcitable. In our recent study of mice treated with oxaliplatin, nNOS‐IR cells increased as a proportion of all neurons, reflecting either selective survival or changes in gene expression.^14^

## Conclusions

Intracellular recording from human enteric neurons is technically challenging; the number of cells that could be recorded in this study was correspondingly small. The technical difficulty may be why relatively few studies have been published using this approach.^22^, ^23^, ^49^ Direct investigation of human enteric neurons, *in vitro*, is invaluable as a tool to translate discoveries in laboratory animals to human patients.^50^, ^51^ To our knowledge, this is the first paper to suggest functional changes to human enteric neurons in a pathological condition, using this direct recording approach. The mechanisms underlying chemotherapy‐induced changes are still to be identified. Future studies in animal models will be invaluable in the attempt to understand the effects of chemotherapy on bowel function.

## Funding

This study is supported by Australian National Health & Medical Research Council Project grant 1032414.

## Disclosure

The authors do not have any potential conflicts to disclose.

## Author Contribution

SEC, SJHB, and KN were responsible for experimental design; SEC and VJ performed all experiments and analysis; SEC drafted the manuscript; KN and SJHB obtained funding and supervised the study. The manuscript was edited and reviewed by all authors.

## References

[1] Goodwin RA , Asmis TR . Overview of systemic therapy for colorectal cancer. Clin Colon Rectal Surg 2009; 22: 251–6. 21037816 10.1055/s-0029-1242465 PMC2796098

[2] Lehky TJ , Leonard GD , Wilson RH , Grem JL , Floeter MK . Oxaliplatin‐induced neurotoxicity: acute hyperexcitability and chronic neuropathy. Muscle Nerve 2004; 29: 387–92. 14981738 10.1002/mus.10559

[3] Miltenburg NC , Boogerd W . Chemotherapy‐induced neuropathy: a comprehensive survey. Cancer Treat Rev 2014; 40: 872–82. 24830939 10.1016/j.ctrv.2014.04.004

[4] Ewertz M , Qvortrup C , Eckhoff L . Chemotherapy‐induced peripheral neuropathy in patients treated with taxanes and platinum derivatives. Acta Oncol 2015; 54: 587–91. 25751757 10.3109/0284186X.2014.995775

[5] Argyriou AA , Cavaletti G , Briani C , Velasco R , Bruna J , Campagnolo M , Alberti P , Bergamo F *et al* Clinical pattern and associations of oxaliplatin acute neurotoxicity. Cancer 2012; 119: 438–44. 22786764 10.1002/cncr.27732

[6] Lucchetta M , Lonardi S , Bergamo F , Alberti P , Velasco R , Argyriou AA , Briani C , Bruna J *et al* Incidence of atypical acute nerve hyperexcitability symptoms in oxaliplatin‐treated patients with colorectal cancer. Cancer Chemother Pharmacol 2012; 70: 889–902. 10.1007/s00280-012-2006-8 23108696

[7] Stringer AM , Gibson RJ , Bowen JM , Logan RM , Ashton K , Yeoh ASJ , Al‐Dasooqi N , Keefe DMK . Irinotecan‐induced mucositis manifesting as diarrhoea corresponds with an amended intestinal flora and mucin profile. Int J Exp Pathol 2009; 90: 489–99. 19765103 10.1111/j.1365-2613.2009.00671.x PMC2768147

[8] Mcquade RM , Bornstein JC , Nurgali K . Anti‐colorectal cancer chemotherapy‐induced diarrhoea: current treatments and side‐effects. IJCM 2014; 5: 393–406.

[9] Stojanovska V , Sakkal S , Nurgali K . Platinum‐based chemotherapy: gastrointestinal immunomodulation and enteric nervous system toxicity. Am J Physiol Gastrointest Liver Physiol 2015; 308: G223–32. 25501548 10.1152/ajpgi.00212.2014

[10] Denlinger CS , Barsevick AM . The challenges of colorectal cancer survivorship. J Natl Compr Canc Netw 2009; 7: 883–93. 19755048 10.6004/jnccn.2009.0058 PMC3110673

[11] Keefe DMK . Gastrointestinal mucositis: a new biological model. Support Care Cancer 2004; 12: 6–9. 14605986 10.1007/s00520-003-0550-9

[12] Furness JB . The enteric nervous system and neurogastroenterology. Nature 2012; 9: 286–94. 10.1038/nrgastro.2012.32 22392290

[13] De Giorgio R , Barbara G , Furness JB , Tonini M . Novel therapeutic targets for enteric nervous system disorders. Trends Pharmacol Sci 2007; 28: 473–81. 17764756 10.1016/j.tips.2007.08.003

[14] Wafai L , Taher M , Jovanovska V , Bornstein JC , Dass CR , Nurgali K . Effects of oxaliplatin on mouse myenteric neurons and colonic motility. Front Neurosci 2013; 7: 30. doi: 10.3389/fnins.2013.00030. 23486839 10.3389/fnins.2013.00030 PMC3594784

[15] Vera G , Castillo M , Cabezos PA , Chiarlone A , Martín MI , Gori A , Pasquinelli G , Barbara G *et al* Enteric neuropathy evoked by repeated cisplatin in the rat. Neurogastroenterol Motil 2011; 23: 370–e163. 21299719 10.1111/j.1365-2982.2011.01674.x

[16] Vera G , López‐Pérez AE , Martínez‐Villaluenga M , Cabezos PA , Abalo R . X‐ray analysis of the effect of the 5‐HT3 receptor antagonist granisetron on gastrointestinal motility in rats repeatedly treated with the antitumoral drug cisplatin. Exp Brain Res 2014; 232: 2601–12. 24798399 10.1007/s00221-014-3954-5

[17] Ozaki A , Sukamoto T . Improvement of cisplatin‐induced emesis and delayed gastric emptying by KB‐R6933, a novel 5‐HT 3 receptor antagonist. Gen Pharmacol 1999; 33: 283–8. 10480662 10.1016/s0306-3623(98)00286-9

[18] Kurian SS , Ferri GL , De Mey J , Polak JM . Immunocytochemistry of serotonin‐containing nerves in the human gut. Histochemistry 1983; 78: 523–9. 6352575 10.1007/BF00496204

[19] De Giorgio R , Bovara M , Barbara G , Canossa M , Sarnelli G , De Ponti F , Stanghellini V , Tonini M *et al* Anti‐HuD‐induced neuronal apoptosis underlying paraneoplastic gut dysmotility. Gastroenterology 2003; 125: 70–9. 12851872 10.1016/s0016-5085(03)00664-4

[20] Wattchow D , Brookes S , Murphy E , Carbone S , De Fontgalland D , Costa M . Regional variation in the neurochemical coding of the myenteric plexus of the human colon and changes in patients with slow transit constipation. Neurogastroenterol Motil 2008; 20: 1298–305. 18662329 10.1111/j.1365-2982.2008.01165.x

[21] Beyer J , Jabari S , Rau TT , Neuhuber W , Brehmer A . Substance P‐ and choline acetyltransferase immunoreactivities in somatostatin‐containing, human submucosal neurons. Histochem Cell Biol 2013; 140: 157–67. 23361835 10.1007/s00418-013-1078-9

[22] Carbone SE , Jovanovska V , Nurgali K , Brookes SJH . Human enteric neurons: morphological, electrophysiological, and neurochemical identification. Neurogastroenterol Motil 2014; 26: 1812–6. 25293378 10.1111/nmo.12453 PMC4265287

[23] Brookes SJ , Ewart WR , Wingate DL . Intracellular recordings from myenteric neurones in the human colon. J Physiol 1987; 390: 305–18. 2895177 10.1113/jphysiol.1987.sp016702 PMC1192182

[24] Linden DR , Sharkey KA , Mawe GM . Enhanced excitability of myenteric AH neurones in the inflamed guinea‐pig distal colon. J Physiol 2003; 547: 589–601. 12562910 10.1113/jphysiol.2002.035147 PMC2342639

[25] Nurgali K , Qu Z , Hunne B , Thacker M , Pontell L , Furness JB . Morphological and functional changes in guinea‐pig neurons projecting to the ileal mucosa at early stages after inflammatory damage. J Physiol 2011; 589: 325–39. 21098001 10.1113/jphysiol.2010.197707 PMC3043536

[26] Carbone SE , Wattchow DA , Spencer NJ , Brookes SJH . Loss of responsiveness of circular smooth muscle cells from the guinea pig ileum is associated with changes in gap junction coupling. Am J Physiol Gastrointest Liver Physiol 2012; 302: G1434–44. 22461022 10.1152/ajpgi.00376.2011

[27] Adelsberger H , Quasthoff S , Grosskreutz J , Lepier A , Eckel F , Lersch C . The chemotherapeutic oxaliplatin alters voltage‐gated Na(+) channel kinetics on rat sensory neurons. Eur J Pharmacol 2000; 406: 25–32. 11011028 10.1016/s0014-2999(00)00667-1

[28] Webster RG , Brain KL , Wilson RH , Grem JL , Vincent A . Oxaliplatin induces hyperexcitability at motor and autonomic neuromuscular junctions through effects on voltage‐gated sodium channels. Br J Pharmacol 2005; 146: 1027–39. 16231011 10.1038/sj.bjp.0706407 PMC1751225

[29] Park SB , Goldstein D , Lin CSY , Krishnan AV , Friedlander ML , Kiernan MC . Acute abnormalities of sensory nerve function associated with oxaliplatin‐induced neurotoxicity. J Clin Oncol 2009; 27: 1243–9. 19164207 10.1200/JCO.2008.19.3425

[30] Krishnan AV , Goldstein D , Friedlander M , Kiernan MC . Oxaliplatin‐induced neurotoxicity and the development of neuropathy. Muscle Nerve 2005; 32: 51–60. 15880395 10.1002/mus.20340

[31] Stein ME , Drumea K , Yarnitsky D , Benny A . A rare event of 5‐fluorouracil‐associated peripheral neuropathy: a report of two patients. Am J Clin Oncol 1998; 21: 248–9. 9626791 10.1097/00000421-199806000-00008

[32] Soares PMG , Mota JMSC , Gomes AS , Oliveira RB , Assreuy AMS , Brito GAC , Santos AA , Ribeiro RA *et al* Gastrointestinal dysmotility in 5‐fluorouracil‐induced intestinal mucositis outlasts inflammatory process resolution. Cancer Chemother Pharmacol 2008; 63: 91–8. 18324404 10.1007/s00280-008-0715-9

[33] Lomax AE , Mawe GM , Sharkey KA . Synaptic facilitation and enhanced neuronal excitability in the submucosal plexus during experimental colitis in guinea‐pig. J Physiol 2005; 564: 863–75. 15774518 10.1113/jphysiol.2005.084285 PMC1464458

[34] Muss HB , Bynum DL . Adjuvant chemotherapy in older patients with stage III colon cancer: an underused lifesaving treatment. J Clin Oncol 2012; 30: 2576–8. 22665545 10.1200/JCO.2012.42.3780

[35] Jaggi AS , Singh N . Mechanisms in cancer‐chemotherapeutic drugs‐ind‐uced peripheral neuropathy. Toxicology 2012; 291: 1–9. 22079234 10.1016/j.tox.2011.10.019

[36] Lin Z , Gao N , Hu HZ , Liu S , Gao C , Kim G , Ren J , Xia Y *et al* Immunoreactivity of Hu proteins facilitates identification of myenteric neurones in guinea‐pig small intestine. Neurogastroenterol Motil 2002; 14: 197–204. 11975720 10.1046/j.1365-2982.2002.00317.x

[37] Qu Z‐D , Thacker M , Castelucci P , Bagyánszki M , Epstein ML , Furness JB . Immunohistochemical analysis of neuron types in the mouse small intestine. Cell Tissue Res 2008; 334: 147–61. 18855018 10.1007/s00441-008-0684-7

[38] Ganns D , Schrödl F , Neuhuber W , Brehmer A . Investigation of general and cytoskeletal markers to estimate numbers and proportions of neurons in the human intestine. Histol Histopathol 2006; 21: 41–51. 16267786 10.14670/HH-21.41

[39] Murphy EMA , Defontgalland D , Costa M , Brookes SJH , Wattchow DA . Quantification of subclasses of human colonic myenteric neurons by immunoreactivity to Hu, choline acetyltransferase and nitric oxide synthase. Neurogastroenterol Motil 2007; 19: 126–34. 17244167 10.1111/j.1365-2982.2006.00843.x

[40] Gorospe M . HuR in the mammalian genotoxic response: post‐transcriptional multitasking. Cell Cycle 2003; 2: 412–4. 12963828

[41] Doxakis E . RNA binding proteins: a common denominator of neuronal function and dysfunction. Neurosci Bull 2014; 30: 610–26. 24962082 10.1007/s12264-014-1443-7 PMC5562623

[42] Hinman MN , Zhou H‐L , Sharma A , Lou H . All three RNA recognition motifs and the hinge region of HuC play distinct roles in the regulation of alternative splicing. Nucleic Acids Res 2013; 41: 5049–61. 23525460 10.1093/nar/gkt166 PMC3643579

[43] Colombrita C , Silani V , Ratti A . ELAV proteins along evolution: back to the nucleus? Mol Cell Neurosci 2013; 56: 447–55. 23439364 10.1016/j.mcn.2013.02.003

[44] Sanna MD , Quattrone A , Mello T , Ghelardini C , Galeotti N . The RNA‐binding protein HuD promotes spinal GAP43 overexpression in antiretroviral‐induced neuropathy. Exp Neurol 2014; 261: 343–53. 24861443 10.1016/j.expneurol.2014.05.017

[45] Wang W , Furneaux H , Cheng H , Caldwell MC , Hutter D , Liu Y , Holbrook N , Gorospe M *et al* HuR regulates p21 mRNA stabilization by UV light. Mol Cell Biol 2000; 20: 760–9. 10629032 10.1128/mcb.20.3.760-769.2000 PMC85192

[46] Mazan‐Mamczarz K , Galbán S , López de Silanes I , Martindale JL , Atasoy U , Keene JD , Gorospe M . RNA‐binding protein HuR enhances p53 translation in response to ultraviolet light irradiation. Proc Natl Acad Sci USA 2003; 100: 8354–9. 12821781 10.1073/pnas.1432104100 PMC166233

[47] Rivera LR , Thacker M , Pontell L , Cho H‐J , Furness JB . Deleterious effects of intestinal ischemia/reperfusion injury in the mouse enteric nervous system are associated with protein nitrosylation. Cell Tissue Res 2011; 344: 111–23. 21305320 10.1007/s00441-010-1126-x

[48] Ince‐Dunn G , Okano HJ , Jensen KB , Park WY , Zhong R , Ule J , Mele A , Fak JJ *et al* Neuronal Elav‐like (Hu) proteins regulate RNA splicing and abundance to control glutamate levels and neuronal excitability. Neuron 2012; 75: 1067–80. 22998874 10.1016/j.neuron.2012.07.009 PMC3517991

[49] Maruyama T . Two types of spike generation of human Auerbach's plexus cells in culture. Neurosci Lett 1981; 25: 143–8. 7279309 10.1016/0304-3940(81)90322-0

[50] Sanger GJ , Broad J , Kung V , Knowles CH . Translational neuropharmacology: the use of human isolated gastrointestinal tissues. Br J Pharmacol 2012; 168: 28–43. 10.1111/j.1476-5381.2012.02198.x PMC3570000 22946540

[51] Mawe GM . Colitis‐induced neuroplasticity disrupts motility in the inflamed and post‐inflamed colon. J Clin Invest 2015; 125: 949–55. 25729851 10.1172/JCI76306 PMC4362261

